# Marine Natural Products: A Source of Novel Anticancer Drugs

**DOI:** 10.3390/md17090491

**Published:** 2019-08-23

**Authors:** Shaden A. M. Khalifa, Nizar Elias, Mohamed A. Farag, Lei Chen, Aamer Saeed, Mohamed-Elamir F. Hegazy, Moustafa S. Moustafa, Aida Abd El-Wahed, Saleh M. Al-Mousawi, Syed G. Musharraf, Fang-Rong Chang, Arihiro Iwasaki, Kiyotake Suenaga, Muaaz Alajlani, Ulf Göransson, Hesham R. El-Seedi

**Affiliations:** 1 Clinical Research Centre, Karolinska University Hospital, Novum, 14157 Huddinge, Stockholm, Sweden; 2 Department of Molecular Biosciences, the Wenner-Gren Institute, Stockholm University, SE 106 91 Stockholm, Sweden; 3 Department of Laboratory Medicine, Faculty of Medicine, University of Kalamoon, P.O. Box 222 Dayr Atiyah, Syria; 4 Pharmacognosy Department, College of Pharmacy, Cairo University, Kasr el Aini St., P.B. 11562 Cairo, Egypt; 5 Department of Chemistry, School of Sciences & Engineering, The American University in Cairo, 11835 New Cairo, Egypt; 6 College of Food Science, Fujian Agriculture and Forestry University, Fuzhou, Fujian 350002, China; 7 Department of Chemitry, Quaid-i-Azam University, Islamabad 45320, Pakistan; 8 Department of Pharmaceutical Biology, Institute of Pharmacy and Biochemistry, Johannes Gutenberg University, Staudingerweg 5, 55128 Mainz, Germany; 9 Chemistry of Medicinal Plants Department, National Research Centre, 33 El-Bohouth St., Dokki, 12622 Giza, Egypt; 10 Department of Chemistry, Faculty of Science, University of Kuwait, 13060 Safat, Kuwait; 11 H.E.J. Research Institute of Chemistry, International Center for Chemical and Biological Sciences (ICCBS), University of Karachi, Karachi 75270, Pakistan; 12 Graduate Institute of Natural Products, Kaohsiung Medical University, Kaohsiung 807, Taiwan; 13 Department of Chemistry, Faculty of Science and Technology, Keio University, 3-14-1 Hiyoshi, Kohoku, Yokohama 223-8522, Japan; 14 Department of Pharmaceutical Biology/Pharmacognosy, Institute of Pharmacy, University of HalleWittenberg, Hoher Weg 8, DE 06120 Halle (Saale), Germany; 15 Pharmacognosy, Department of Medicinal Chemistry, Uppsala University, Box 574, SE-75 123 Uppsala, Sweden; 16 Department of Chemistry, Faculty of Science, Menoufia University, 32512 Shebin El-Koom, Egypt; 17 College of Food and Biological Engineering, Jiangsu University, Zhenjiang 212013, China; 18 Al-Rayan Research and Innovation Center, Al-Rayan Colleges, 42541 Medina, Saudi Arabia

**Keywords:** marine, plants, microorganism, antitumor, anticancer, cytotoxic, clinical trials, drugs

## Abstract

Cancer remains one of the most lethal diseases worldwide. There is an urgent need for new drugs with novel modes of action and thus considerable research has been conducted for new anticancer drugs from natural sources, especially plants, microbes and marine organisms. Marine populations represent reservoirs of novel bioactive metabolites with diverse groups of chemical structures. This review highlights the impact of marine organisms, with particular emphasis on marine plants, algae, bacteria, actinomycetes, fungi, sponges and soft corals. Anti-cancer effects of marine natural products in in vitro and in vivo studies were first introduced; their activity in the prevention of tumor formation and the related compound-induced apoptosis and cytotoxicities were tackled. The possible molecular mechanisms behind the biological effects are also presented. The review highlights the diversity of marine organisms, novel chemical structures, and chemical property space. Finally, therapeutic strategies and the present use of marine-derived components, its future direction and limitations are discussed.

## 1. Introduction

**Figure 1:**
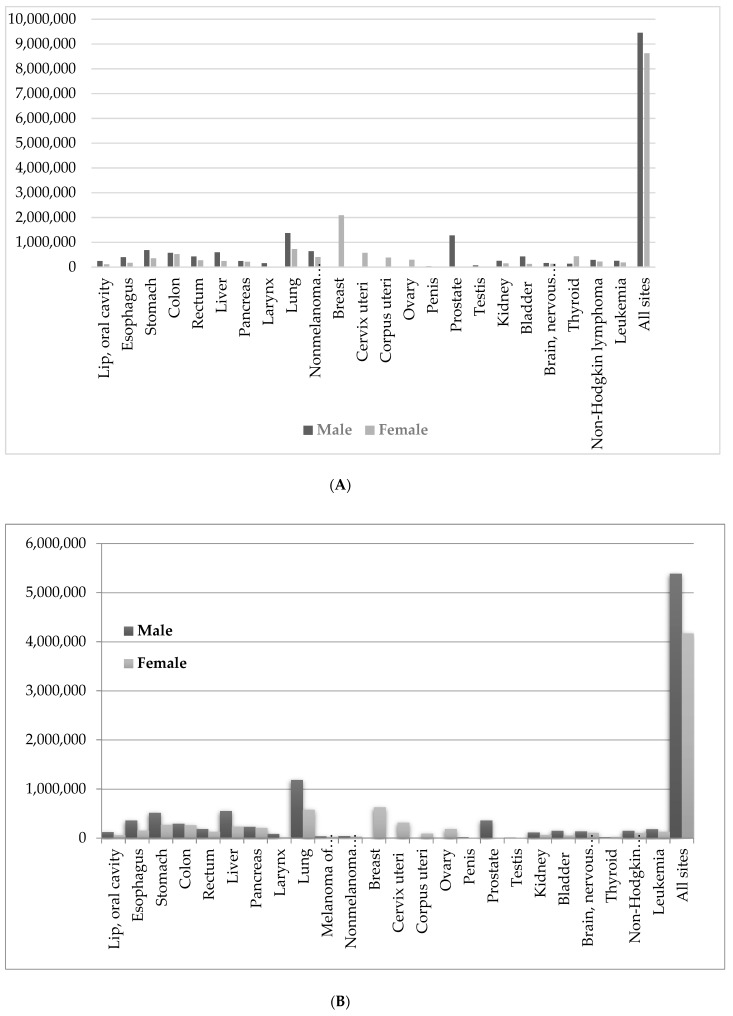

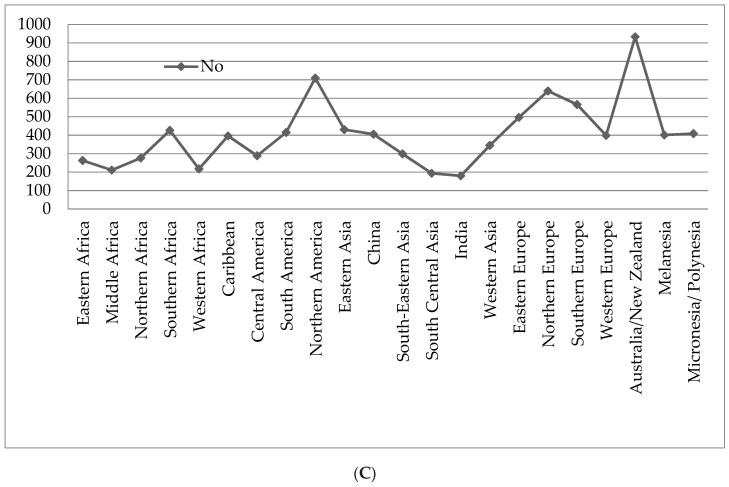
(**A**) Estimated new cancer cases in the worldwide based on Global Cancer (GLOBOCAN)2018. (**B**) Estimated cancer death in the worldwide based on GLOBOCAN 2018. (**C**) Estimated number of new cancer cases in different world areas based on GLOBOCAN 2018.

Cancer remains one of the most life-threatening diseases worldwide. In 2018, approximately 18 million new cases of cancer reported globally, resulting in approximately 10 million deaths [1]. Figure 1A–C show the estimated new cancer cases of different body tissues, the estimated cancer deaths, and estimated number of new cancer cases in different world areas, respectively. Over 1,000,000 new cases and 65,000 deaths are estimated globally, with an incidence rate around two times higher among men than women. Nonmelanoma skin cancer (NMSC) is the most frequently diagnosed cancer in North America, and in Australia, New Zealand, the countries with the highest incidence rates worldwide in men and women, respectively [1]. The highest incidence rates were reported with increased risk is associated with age, and an unhealthy lifestyle [2]. The incidence of cancer continues to increase due to environmental changes and life style modernization [3]. Lung and breast cancer are the most frequently diagnosed cancers worldwide and are the leading causes of cancer-related death in men and women, respectively. Meanwhile, the predisposition factors of cancer could be both external (tobacco, chemicals, radiation, and infectious organisms), and internal (genetic predispositions, immune conditions) [2].

The progress in biological, immunotherapy and the substantial improvements in modern drug design and manufacturing have made the discovery of a cure for cancer a feasible goal [4]. Cure and prolonged survival have already been achieved for a number of human malignancies, such as lymphomas, testicular cancer, and childhood lymphoblastic leukemia [5,6,7]. Despite the significant advances of current therapies [8,9], multiple side effects have been reported with chemotherapy [10], motivating the search for other effective cure with fewer side effects [10]. Natural products represent an available source of new drugs, drug leads and chemical entities [11,12]. Approximately 80% of the approved chemotherapeutic drugs [13], and more than half of all drugs are based on bioactive natural products [14]. Eighty-seven percent of human diseases, including cancer, are treated using natural products [15]. Natural bioactive molecules exhibit cytotoxic effects by attacking macromolecules expressed by cancer cells, such as those in oncogenic signal transduction pathways [16]. A significant number of marine-derived metabolites act as antitumor agents via potent growth inhibition of human tumor cells both in vitro, in vivo (in murine) models and in cancer clinical trials [13,17]. Advanced technology and extensive research on marine natural products have led to the discovery of a new generation of anticancer drugs currently used in clinical trials [6].

Marine have great potential for discovery of new entities that can aid in the prevention and treatment of cancer. Marine emerged in the late 19th century. After 1980, biotechnology emerged as a field that provided direction to the study of marine, aiming at applications such as drug development. This research is still ongoing using advanced tools [18]. Given the great potential of marine natural product scaffolds, there is an increasing interest for exploiting this diversity and complexity for rational drug discovery [16]. Natural products, in general, have been a prime source of compounds for the treatment of many forms of cancer, and offer a promising opportunity for evaluation of not only new chemical classes of anticancer agents but also novel and potentially relevant mechanisms of action [19].

## 2. Nature and Cancer Chemotherapy

Over the past 50 years, emerging evidence has shown that many natural products derived from plants and microbes of marine origin (Table 1), exhibit beneficial effects in the prevention and treatment of cancer, i.e., cytarabine, eribulin mesylate, brentuximab vedotin, and trabectidine are marine-based drugs used against leukemia, metastatic breast cancer, soft tissue sarcoma and ovarian cancer [20,21]. Not only the marines represent the main source for anticancer drugs but also there are other vital sources like as plants, animals, invertebrates and terrestrial microbes, for example Taxol; is an antineoplastic drug obtained from the bark of the Western Yew tree (*Taxus brevifolia* L., Taxaceae), proved to be useful in the treatment of breast cancer [22], in addition to the active complex alkaloid compounds such as Vincristine and Vinblastine which are present in Vinca herb. They have proven effective agents against childhood leukemia and Hodgkin’s disease (a cancer of the lymph nodes), choriocarcinoma, respectively [23].

**Figure 2:**
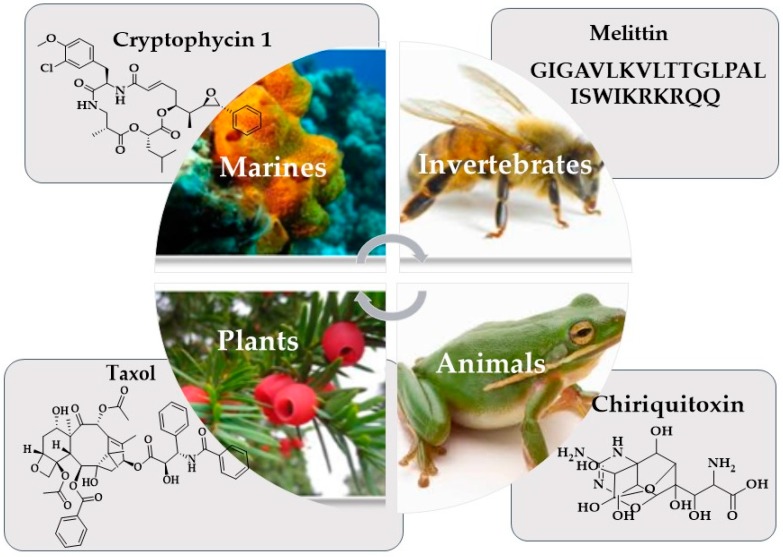
Natural sources for cancer control.

Although the National Cancer Institute provides researchers with the resources needed to better elucidate the role of food and nutrients in cancer prevention, cancer chemoprevention using marine natural compounds [16,24] has not been investigated in-depth and the preclinical and clinical data for this strategy remain scant, in spite of the robust chemical rationale [16]. Many other compounds with anticancer properties have been isolated and developed from various biological resources, such as plants, microbes and marine organisms (Figure 2). Consequently, a large number of natural products are in preclinical investigations, and 13 natural products isolated from marine organisms are being tested in different phases of clinical trials, highlighting the potential of marine natural compounds [25]. A focused, combinatorial approach would has been suggested to accelerate the development of new anti-cancer drugs from marine resources with increased efficiency and fewer side effects [26].

## 3. Marine Organisms and Cancer Chemotherapy

Oceans cover over 70% of the earth. The total global biodiversity is estimated to include ca. 500 × 10^6^ species of prokaryotic and eukaryotic organisms. The marine environment is indeed an exceptionally diverse reservoir of life, containing nearly 250,000 described species [27,28]. Among marine organisms, 3.7 × 10^30^ microorganisms have been discovered in marine environments [29], 99% of all bacteria cannot be cultured but can synthesize many fascinating natural products that are potential drug leads [30]. This extraordinary chemical and pharmacological scope of marine organisms could be attributed for the necessity to produce secondary metabolites as defense tools to survive in extreme environments; of temperatures, salinity, pressure and to resist predators.

Since ancient times, marine flora has been used for medicinal purposes worldwide: in India, China, the Near East, and Europe [31]. From then till now, less than 5% of the deep sea has been explored, and less than 0.01% of the deep-sea floor has been sampled in detail [32]. The Caribbean sponge (*Cryptotethya crypta*) was the first marine organism to be investigated in detail chemically [33], and extensive phytochemical studies on pure compounds from this organism were performed from 1950 to 1960, before the identification of cytosine arabinoside (ara-C) [34,35,36]. Furthermore, some marine organisms, such as microflora (bacteria, actinobacteria, cyanobacteria and fungi), microalgae, macroalgae (seaweeds) [31], invertebrate animals [37,38] sponges, soft corals, sea fans, sea hares, nudibranchs, bryozoans, tunicates, etc. [2], have been investigated for cancer control [39,40]. The bio-active molecules impact has been evaluated against various cancer types in clinical trials [41,42,43,44,45]. Additionally, with the ongoing advancement in marine chemistry, new tools have been employed, e.g., metabolomics, to examine marine products from different perspectives [31].

## 4. Characterization of Marine Metabolites

The major obstacles for better understanding of marine metabolites chemistry and composition are sampling difficulties. Sufficiently large quantities are required for detailed analyzes and resolution of the instrumental and bioassay approaches used. For modern analytical methods in mass spectrometry (MS), and nuclear magnetic resonance (NMR) spectroscopy [46], sub mg, or low micro gram, amounts may be enough for full structure elucidation. The marine community has used MS for the past three to four decades. A separation method is commonly used together with MS to enhance resolution and selectivity, in hyphenated techniques such as LC-MS, LC-MS/MS, GC-MS, pyrolysis-GC-MS, and direct temperature-resolved MS (DT-MS) methods [47,48,49,50]. MS has been mainly used in past studies for identifying and quantifying the specific fractions or trace components within the marine organisms, and identification is then aided by MS/MS analysis, as exemplified by the identification of hierridin B from a marine cyanobacterium *Cyanobium* spp. strain [51]. There is no doubt that NMR is the most widely used technique for structural characterization of molecules, i.e., acremines P was isolated from a marine-derived strain of *Acremonium persicinum* and identified using NMR techniques [52]. Nevertheless, the lower sensitivity level of NMR compared to MS hinders the identification of metabolites present at trace levels or low amounts. Although originally used for small, relatively simple organic compounds, it has gained widespread popularity as a method for marine´s metabolites fingerprinting organism [53,54]. However, although LC-HRMS is extremely sensitive and can detect compounds present at very low quantities, there are certain classes of compounds that cannot be detected by MS; they may not form ions at all, or ion formation may be suppressed or they are not able to be eluted from the column to be detected. NMR, on the other hand, has no separation step and therefore provides a snapshot of the metabolome of the sample. It is less sensitive than MS, but more robust and reproducible with universal feature in metabolites detection, all of which allowing for comparison of results from different datasets or running at different time periods. Also, coupling of metabolomics to other “omics” technologies, i.e., genomics could aid in better correlation of marine metabolome in relation to its genotype. [55]. These analytical techniques are also used to elucidate the function/mode of action of metabolites. For example, functional metabolomics was employed to reveal metabolic alterations associated in MCF-7 breast cancer cells exposed to the alkaloid ascididemin [56].

## 5. From Marine Organisms to Anticancer Drugs

**Figure 3:**
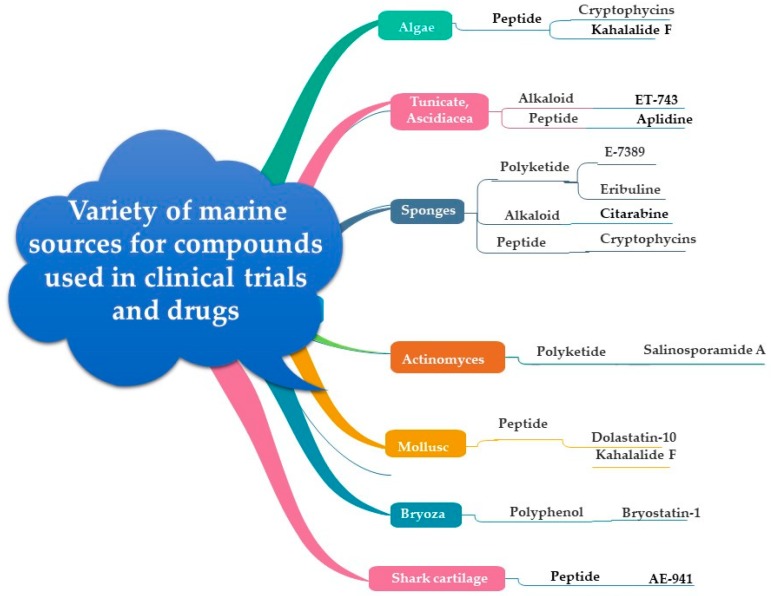
Marine drugs and compounds used in clinical trials, its sources and chemical classes.

There are more than 22,000 known microbial secondary metabolites, 70% of which are produced by actinomycetes, 20% by fungi, 7% by *Bacillus* spp. and 1–2% by other bacteria [57]. It should be noted that generally 10% of all currently known biologically active natural products are of microbial origin. There are few examples of marine antineoplastic agents that have reached clinical phase trials. For instance, bryostatin 1, ET-743 and dolastatin 10. The bryostatin 1 has recently entered phase II clinical trial against melanoma, non-Hodgkin’s lymphoma, renal cancer and colorectal cancer [58,59,60]. The biological effect of bryostatin 1 is mediated via the promotion of normal growth of bone marrow progenitor cells [61]. Moreover, ET-743, a tetrahydroisoquinilone alkaloid isolated from tunicate *Ecteinascidia turbinata* entered phase I clinical trials [62], since it exerts anti-proliferative effects by selective alkylation of guanine residues in the DNA minor groove [63], whereas dolastatin 10, a member of a peptide family isolated from the mollusk *Dolabella auricularia*, reached phase II clinical trials [64], based on its inhibition of microtubule assembly, which eventually leads to metaphase arrest in the cell cycle [65,66], (Table 2, Figure 3).

### 5.1. Marine Plants

Marine plants have rarely been discussed in the literature as a distinct and self-contained group. These plants have traditionally been treated either as the poor relations of marine animals in courses and texts on marine biology or as examples of particular groups of algae, where the essential ‘marine-ness’ of marine plants tends to disappear among the taxonomic and morphological parallels with freshwater algae. Over 90% of marine plant species are algae [67]. Because there is great chemical diversity in marine plants, including marine algae and mangroves, products isolated from these plants have been shown to possess antibacterial, antifungal, analgesic, anti-inflammatory, cytotoxic, hypotensive, and spasmogenic activities [68,69].

#### 5.1.1. Macroalgae (Seaweed)

Macroalgae have long been recognized as food, functional food and potential drug sources [70]. Also known as seaweed, multicellular macroalgae contain numerous pharmacologically important bioactive elements to include carotenoids, dietary fiber, protein, essential fatty acids, vitamins (A, B, B_12_, C, D, E), and minerals such as Ca, P, Na, and K [70,71,72,73], in addition to polyphenols [74,75]. An alcoholic extract of the red alga *Acanthophora spicifera* was supplemented to mice treated with Ehrlich’s ascites carcinoma cells, and to exhibit anti-tumor activity at an oral dose of 100 and 200 mg/kg [76]. Similarly, an extract of the brown seaweed *Sargassum thunbergii* displayed antitumor activity against transplanted tumor such as sarcoma 180 and Ehrlich solid carcinoma (in vivo) [77]. The anti-proliferative effect of fucoidan, isolated from *Ascophyllum nodosum* was demonstrated against sigmoid colon adenocarcinoma cells (COLO320 DM), in comparison to fibroblasts (hamster kidney fibroblast CCL39) [78]. Caulerpenyne from *Caulerpa* sp. algea attributed to anticancer and antiproliferative effects against neuroblastoma cell line through induction of cells inhibition proliferation with an IC_50_ of 10 µM [79]. Condriamide-A, isolated from *Chondria* sp., showed a cytotoxic effect at a dose of 0.5 µg/mL against KB cells and 5 µg/mL against LOVO cells (colon cancer) [80]. Two compounds isolated from *Cystophora* sp., namely, meroterpene and usneoidone, have demonstrated antitumor properties [79,81,82,83]. Sulfated polysaccharides purified from the brown alga *Eclonia cava* selectively and dose-dependently suppressed the proliferation of murine colon carcinoma (CT-26) and human leukemic monocyte lymphoma (U-937) cell lines [84]. Equally important, stylopoldione, a potent cytotoxic metabolite isolated from *Stypodium* sp., disrupted mitotic spindle formation functioning via inhibiting synchronous cell division using urchin egg assay (*Strongylocentrotus purpuratus* Stimpson) at ED_50_ = 1.1 µg/mL, and to inhibit cells cleavage via inhibition of tubulin polymerization [85].

#### 5.1.2. Microalgae

Cyanobacteria, also known as blue-green algae, are prolific sources of more than 400 novel metabolites, particularly unique, biologically active peptide and polyketide metabolites [86], effective at either killing cancer cells by inducing apoptotic death or affecting cell signaling via activation of the protein kinase c family [31]. Approximately half of the 41 screened strains of cyanobacteria exhibited the ability to cause cancer cell death [87]. Two cyanobacteria-derived anti-microtubule agents, i.e., dolastatin 10 and curacin A, have been clinically evaluated for the treatment of cancer and to serve as lead structures for the synthesis of a number of synthetic analogs/derivatives [88]. Calothrixins A and B, are pentacyclic metabolites isolated from *Calothrix cyanobacteria* with anticancer potent activity against human HeLa cancer cells in a dose-dependent manner at an IC_50_ of 40 and 350 nM, respectively (in vitro studies) [89]. Ulithiacyclamide and patellamide, produced by cyanobacteria *Prochloron* spp. and *Lissoclinum patella* [90,91,92], exhibited potent cytotoxic activity against a human nasopharyngeal carcinoma cell line at IC_50_ value of 17 and 3000 ng/mL, respectively [93]. Borophycin, a boron-containing metabolite isolated from marine cyanobacterial strains of *Nostoc linckia* and *Nostoc spongiaeforme* var. *tenue* [94], attributed potent cytotoxicity against human epidermoid carcinoma (LOVO) and human colorectal adenocarcinoma (KB) cell lines [95]. Potent cytotoxicity was displayed by cryptophycin 1, isolated from *Nostoc* sp. GSV 224, against tumor cells in vitro (human tumor cell lines (KB and LOVO with IC_50_ = 0.005; 0.003 ng/mL)) and in vivo (human solid tumors (colon adenocarcinomas, pancreatic ductal adenocarcinoma and mammary adenocarcinoma) [96,97]. Largazole represented a unique chemical scaffold derived from *Symploca* spp. with impressive anti-proliferative activity [98]. The parental compound, apratoxin A, isolated from a strain of *Lyngbya boulloni*, exhibited cytotoxicity against adenocarcinoma [99]. Coibamide A, a promising anti-cancer agent with a new potential mechanism of action, derived from a strain of *Leptolyngbya*, exhibited significant cytotoxicity against NCIH460 lung and mouse neuro-2a cells (LC_50_ < 23 nM) [100]. Cyanobacteria produce a family of antitumor agents known as cryptophycins, which interfere with tubulin assembly [101]. Scytonemin, is a protein serine/threonine kinase inhibitor of the cell division cycle 25C (cdc25C) in a dose-dependent manner with an IC_50_ of 2.3 µM where significant inhibition was observed at concentrations as low as 300 nM [102]. Scytonemin is present in the extracellular sheaths of different genera of aquatic and terrestrial blue-green algae. This compound regulates mitotic spindle formation as well as enzyme kinases involved in cell cycle control, and to also inhibit the proliferation of human fibroblasts and endothelial cells [103]. Curacin A, isolated from the organic extracts of Curacao collections of *Lyngbya majuscule*, is an exceptionally potent anti-proliferative agent that inhibited tubulin polymerization and also exhibited selective inhibitory activity against leukemia and Burkitt lymphoma cell lines (IC_50_ = 9 nM and 200 nM) [104,105]. Apratoxins represent are another class of cyanobacterial compounds that inhibited a variety of cancer cell lines at nanomolar dose levels.

Various strains of cyanobacteria exhibited apoptotic activity against acute myeloid leukemia cells without affecting non-malignant cells, e.g., hepatocytes and cardiomyoblasts [106]. Based on modern research, cultured benthic cyanobacteria from temperate marine environments provide a promising, under-exploited source for novel drugs against leukemia [107]. Nevertheless, there are some compounds isolated from marine sources, not been yet applied in clinical trials like as calothrixins A, B, ulithiacyclamide, patellamide, borophycin, largazole, etc. This all compound shows anticancer activity against various types of cancer cells with different mechanisms hence we recommend further investigation of their potential biological activities and clinical uses.

### 5.2. Marine Bacteria

Marine *Pseudomonas*-derived bioactive substances are diverse and include pyrroles, pseudopeptides, pyrrolidinedione, phloroglucinol, phenazine, benzaldehyde, quinoline, quinolone, phenanthrene, phthalate, andrimid, moiramides, zafrin and bushrin [108]. Some of these bioactive compounds are antimicrobial agents, whereas dibutyl phthalate and di-(2-ethylhexyl) phthalate have been reported to be cathepsin B inhibitors [109]. Discodermolide, bryostatins, sarcodictyin, and eleutherobin are among the most effective anticancer drugs produced mainly by marine bacteria [31,110].

In vivo, *Lactobacilli* and *Noctiluca scintillans* exhibited chemopreventive effects against colon cancer and melanoma cancer [104], respectively. *Lactobacilli* has the ability to reduce the activities of azoreductase, nitroreductase, and β-glucuronidase enzymes in the diet of rats as these dietary components were able to reduce the standard level of enzymes in the intestinal tract thus *Lactobacilli* suggestive that it could lessen the incidence of colon cancer development [111,112]. Probiotic bacteria, such as *Lactobacilli* and *Bifidobacteria*, produce anticancer substances [113]. The marine-derived *Halomonas* spp. strain GWS-BW-H8hM was reported to inhibit the growth of HM02 (gastric adenocarcinoma), HepG2 (hepatocellular carcinoma) and MCF7 cell lines to induce apoptosis via cell cycle arrest compared to actinomycin D [114,115]. Highly heterogeneous polymers, i.e., exopolysaccharides (EPSs) and sulfated EPSs isolated from *H. stenophila* inhabiting a hypersaline environment have also been reported for their pro-apoptotic effects on T-leukemia cells. Only tumor cells were found susceptible to apoptosis induced by the sulphated EPS (B100S), whilst primary T cells were resistant [116]. The isolation of cytotoxic hydroxyphenylpyrrole dicarboxylic acids, i.e., 3-(4-hydroxyphenyl)-4-phenylpyrrole-2,5-dicarboxylic acid (HPPD-1), 3,4-di-(4-hydroxy-phenyl) pyrrole-2,5-dicarboxylic acid (HPPD-2) and the indole derivatives 3-(hydroxyacetyl)-indole, indole-3-carboxylic acid, indole-3-carboxaldehyde, and indole-3-acetic acid, from a marine *Halomonas* sp. has also been reported [117]. Both HPPD-1 and HPPD-2 exhibited potent antitumor activities via the inhibition of 12-*O*-tetradecanoylphorbol-13-acetate (TPA) induced activation of Epstein–Barr virus early antigen. The inhibitory effect of HPPD-2 was more potent with respect to HPPD-1 at all tested dose ratios: for instance, 32 nmol (1.1 × 10^−2^ mg mL^−1^ in DMSO; 1000% ratio to TPA) of HPPD-2 led to 90% inhibition of TPA-induced activation of EBV-EA (residual activation 10.8%) [118]. The two most active extracts were obtained from isolates of *Sulfitobacter pontiacus* (P1-17B (1E)) and *Halomonas axialensis* (P5-16B (5E)), that inhibited the growth of HeLa and DU145 cells by 50–70%. The cytotoxic activity observed in isolates P1-37B and P3-37A (Halomonas) could be attributed to the aforementioned cytotoxic compounds from *Halomonas* spp. extracts prepared from *Chromohalobacter salexigens* (P3-86A, K-30, P3-86B (2), *H. meridian* (P3-37B), *Idiomarina loihiensis* (P3-37C) and *C. israelensis* (K-18) were found to be the most active at inducing apoptosis in HeLa cells [115].

### 5.3. Marine Actinomycetes

Marine actinomycetes include members of the genera *Dietzia*, *Rhodococcus* [119], *Streptomyces* [120], *Salinispora* [121,122,123], and *Marinispora* [120,121,122,123]. Actinomycetes are undoubtedly the largest producers of secondary metabolites among marine microorganisms [124]. Actinomycete-isolated secondary metabolites account for *ca*. 45% (~10,000 compounds) of the total known anti-microbial metabolites. Of these actinomycete-derived compounds, 75% were derived from *Streptomyce* whereas 25% were derived from rare actinomycetes [125,126].

Actinomycetes, Streptomyces and Micromonosporaceae are good candidates for the isolation of potent growth-inhibiting compounds and novel antitumor agents [31,106,126,127,128]. In the exploration of marine-derived actinomycetes as sources of antitumor compounds, lucentamycins A-D, which are 3-methyl-4-ethylideneproline-containing peptides were isolated from *Nocardiopsis lucentensis* (strain CNR-712). Lucentamycins A and B exhibited significant in vitro cytotoxicity against HCT-116 human colon carcinoma using MTS assay with IC_50_ = 0.20 and 11 µM, respectively [129]. Thicoraline, a depsipeptide isolated from *Micromonospora marina*, displayed cytotoxic activity against both LOVO and SW620 human colon cancer cell lines with IC_50_ of 15 nM and 500 nM, respectively in vitro. 

Thiocoraline cytotoxic action was found mediated via an arrest in G1 phase of the cell cycle and a decrease in the rate of S phase progression towards G2/M phases, as assessed using bromodeoxyuridine/DNA biparametric flow cytometric analysis [117]. Trioxacarcins A-C extracted from *Streptomyces* species showed high anti-tumor activity against lung cell line with IC_50_ ranging from 0.1, 6.0, 0.003 to 0.26 ng/mL, respectively [130]. Mansouramycin A-D and 3-methyl-7-(methylamino)-5,8-isoquinolinedione, from the marine-derived Mei37 isolate of *Streptomyces* sp., exhibited significant cytotoxicity with a great degree of selectivity for non-small-cell lung cancer, breast cancer, melanoma, and prostate cancer cells [131]. Macrodiolide tartrolon D, extracted from *Streptomyces* sp. MDG-04-17-069, exhibited strong cytotoxic activity against three human tumor cell lines, *viz*., lung (A549), colon (HT29), and breast (MDA-MB-231) cancer (GI_50_ = 0.16, 0.31 and 0.79 µM) compared to doxorubicin as a standard [132]. Salinosporamide A, another compound isolated from a marine-derived actinomycete, is a highly potent irreversible inhibitor of the 20S proteasome that exhibited selective cytotoxic effect against A-549, HL-60, BEL-7402 and P388 cell lines at IC_50_ = 0.13, 0.28, 7.5, 35.0 µM, respectively, and was tested in clinical trials as an anticancer agent (Table 2) [133,134].

### 5.4. Marine Fungi

Marine-derived fungi represent a rich and promising source of novel anticancer agents [135,136]. Higher fungi (basidiomycetes), endophytic fungi and filamentous fungi from marine habitats yielded biologically active principal compounds, such as leptosphaerin,, leptosphaerolide (and its *O*-dihydroquinone derivative), and leptosphaerodione from the lignicolous fungus *Leptosphaeria oraemaris* (Pleosporaceae) [137,138,139]. Antioxidative effects against free radical reactions associated with atherosclerosis, dementia, and cancer were exhibited by (I) acremonin A from *Acremonium* spp. [140], and (II) a xanthone derivative from *Wardomyces anomalus* [141]. The topo I isomerase inhibitor (+)-3,3,7,7,8,8-hexahydroxy-5,5-dimethylbianthraquinone, isolated from both *Aspergillus candidus* and *A. terreus*, showed in vitro cytotoxic and anticancer effects [142,143]. Aspergiolide A, isolated from the marine filamentous fungus *A. glaucus*, contributed to the cytotoxicity against the A-549, HL-60, BEL-7402, and P388 cell lines [144], whereas alkaloids isolated from *Penicillium* spp. derived from deep-ocean sediment displayed antitumor activities. Two new alkaloids meleagrin analogs, meleagrin D and E, and two new diketopiperazines, roquefortine H and I, showed cytotoxic activity toward A-549 and HL-60 cells via apoptosis and arrested the cell cycle at G2/M phase [145]. The anticancer activity of 14 anthracenedione derivatives of secondary metabolites of the mangrove endophytic fungi *Halorosellinia* spp. and *Guignardia* spp. has been reported [146]. The 14 anthracenedione derivatives were found to function via apoptosis induction [142].

### 5.5. Marine Sponges

These organisms contributed to nearly 30% of all-natural products discovered to date [147]. The initial discoveries from marine sponges led to the belief that it would not be long before true marine-derived drugs would reach the market. One successful example is the discovery and identification of spongothymidine and spongouridine from the Caribbean sponge *Tethya crypta*. These compounds were found to possess antiviral activity and synthetic analogs studies eventually led to the development of cytosine arabinoside (AraC) as a clinically anticancer agent [148]. Eribulin, a truncated synthetic version of halichondrin B, derived from the sponge *Halichondria okadai* [149], has clinically potential activity against pretreated metastatic breast cancer cells [150,151].

### 5.6. Soft Corals

*Sarcophyton* is one of the most widely distributed soft coral genera in the tropical and sub-tropical oceans, and approximately 30 species from this genus have been collected and tested for the presence of bioactive secondary metabolites, i.e., fatty acids (arachidonic, eicosapentaenoic, docosahexaenoic acids) that showed cytotoxic activity against brine shrimp in dose-dependent manner (LC_50_ of 96.7 ppm) [152,153]. Among the most important components of soft coral are cembranoids, which are present at high concentrations (up to 5% dry weight). Cembranoids have an impact on biological activities, i.e., ichthyotoxic, cytotoxic, anti-inflammatory, and antagonistic activity. In vitro cytotoxicity testing showed that furano-cembranoids and decaryiol isolated from *Nephthea* spp. and *Sarcophyton cherbonnieri* are effective against several tumor cell lines (gastric epithelial, breast and liver) (with GI_50_ values ranging from 0.15 to 8.6 µg mL^−1^) via arrests the cell cycle in the G2/M phase [154]. In addition, crassumolide C isolated for the first time from (the soft coral *Lobophytum crissum*) was found to inhibit the accumulation of the pro-inflammatory proteins iNOS and COX-2 at 10 µM, as well has a cytotoxic effect toward Ca9-22 cancer cells with IC_50_ of 1.7 µg mL^−1^ compared to doxorubicin; appositive control [155].

## 6. Bioactive Constituents of Marine Organisms

Polyphenols, polysaccharides, and alkaloids are among the highly active, biologically potent and predominant anticancer compounds isolated from marine organisms.

### 6.1. Polyphenols

**Figure 4:**
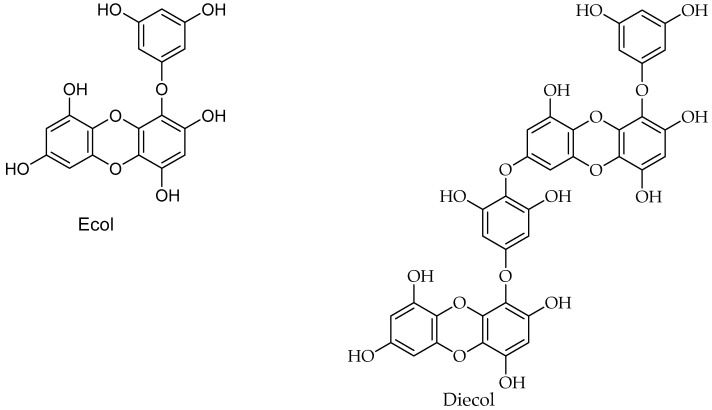

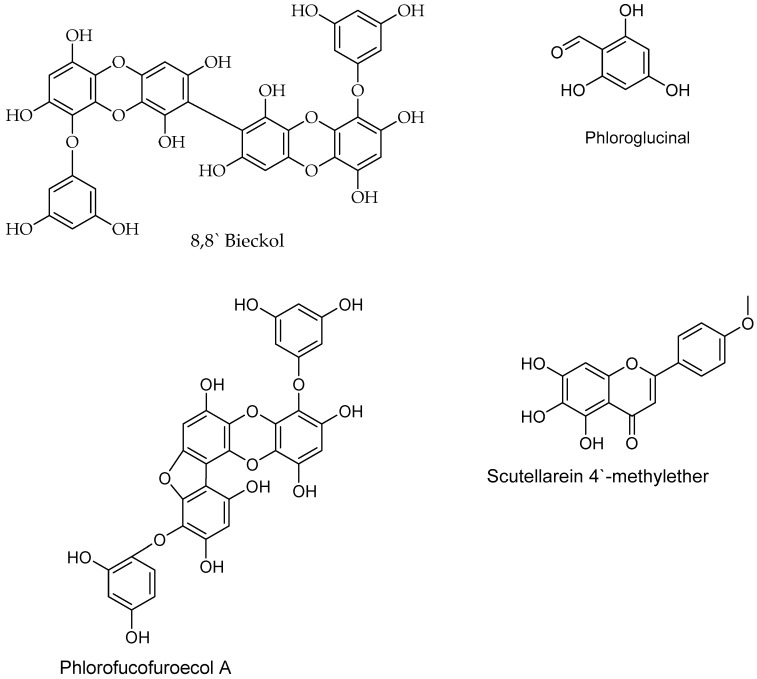
Polyphenolic anti-cancer compounds of marine organisms.

Polyphenols (Figure 4; Table 1), are categorized into phenolic acids, flavonoids, tannins, catechin, anthocyanidins, epigallocatechin, lignin, epicatechin, epigallate, and gallic acid [74,75]. Polyphenolic compounds are known for their potential to reduce the mitotic index and decrease the levels of cellular proteins needed for cancer cell proliferation and colony formation. For example, scutellarein 4’-methyl ether exhibited anticancer effects in vitro and in vivo due to its cytotoxic activities. In addition to anticancer effects, the phenols exhibited anti-inflammatory activity, antiviral effects, and inhibited the human platelet aggregation [156,157,158,159,160]. *Palmaria palmata*, an edible seaweed, is rich in polyphenols with potential antioxidant and anticancer properties [161,162,163]. These polyphenols showed metabolic inhibition of xenobiotic-metabolizing enzymes [164], leading to alteration of the mitotic process in the telophase and thus disruption of cell division [159].

### 6.2. Polysaccharides

**Figure 5:**
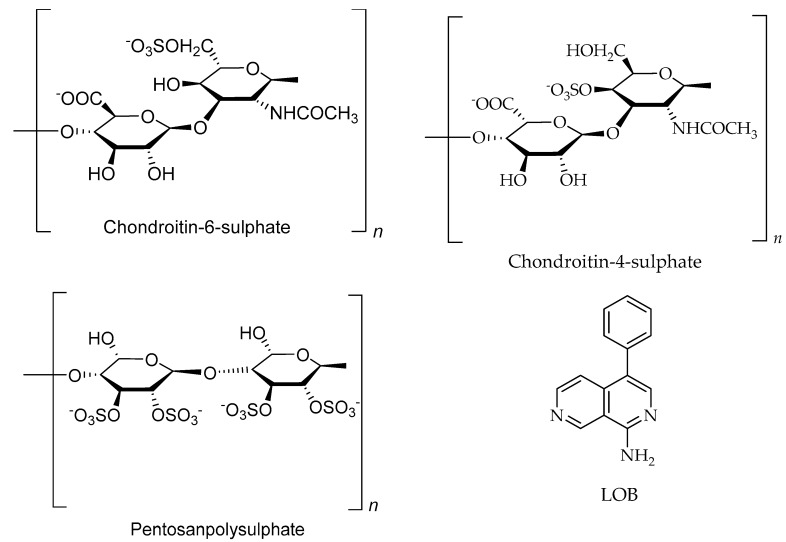

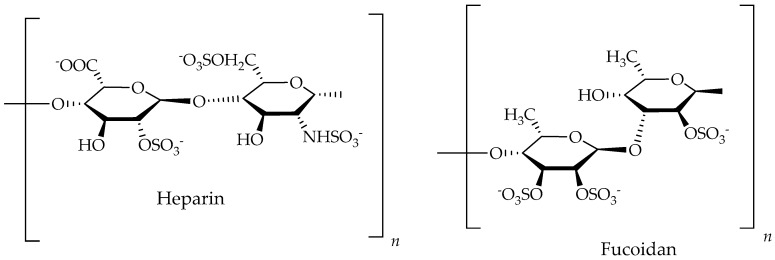
Polysaccharides of marine organisms against cancer.

The other potent group of compounds that is abundantly present in several marine organisms is polysaccharides (Figure 5; Table 1), primarily alginates, agar, and carrageenans [31]. The main mechanism of action of polysaccharides cytotoxic effect is the activation of the innate immune system [165,166,167,168], leading to attraction of macrophages and natural killer cells to the target site and production of tumoricidal cytokines [166,169,170,171]. A sulfated polysaccharide isolated from a marine *Pseudomonas* spp. (B-1) filtrate induced apoptosis of human leukaemic cells (U937) [172], whereas pancreatic islet carcinoma apoptosis was observed with PI-88, a sulfated oligosaccharide [173]. Glycosaminoglycans are sulfated internally and thus induced murine melanoma cell apoptosis by altering transcription [174]. Fucoidan, a sulfated polysaccharide (sulfated L-fucose) from the brown algal cell wall [175,176,177], was able to modulate atherosclerosis, angiogenesis, and metastasis [178], when tested against human lymphoma HS-Sultan cell line. This effect was explained by the consequent activation of caspase-3 and down regulation of the kinase pathway [179]. Fucoidan can disrupt heparansulfate-growth factor/cytokine complexes and act as a substitute for cell surface heparansulfates by stabilizing the interaction between growth factors and their receptors [31].

### 6.3. Alkaloids

**Figure 6:**
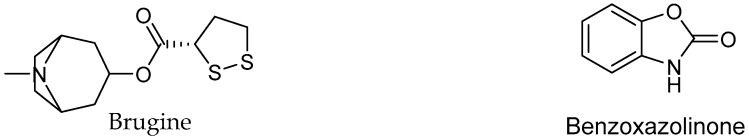
Alkaloids from marine organisms for anticancer.

Alkaloids derived of marine origin are divided into four groups, namely, indoles, halogenated indoles phenylethylamines, and other alkaloids (Figure 6; Table 1), most of which belong to phenylethylamines and indoles [31]. Two derivatives, namely, lophocladine A and lophocladine B, have been isolated from the red alga *Lophocladia* spp. [180]. Similarly, the presence of alkaloids, e.g., acanthicifolin, brugine and benzoquinones, in *Acanthus illicifolius*, *Bruguiera sexangula*, and *Kandelia candel* has been reported [31]. “Rhizophrine” is a major alkaloid constituent of the leaves of *Rhizophora mucronata* and *R. stylosa*, species of mangrove found on coasts and river banks in East Africa and the Indo-Pacific region. The growth inhibitory activity of these compounds has been successfully demonstrated using various cancer cell lines.

### 6.4. Peptides

**Figure 7:**
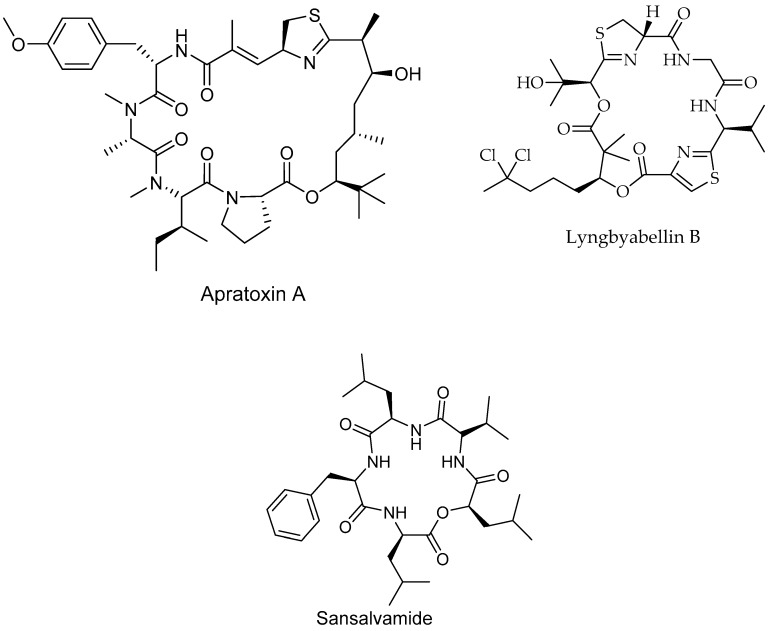
Anticancer peptides from marine organisms.

Different types of peptides (Figure 7; Table 1), have been isolated from a wide variety of marine flora. In the last decade, ca. 2500 new peptides with anti-proliferative activity have been identified [110]. Purified peptides have exhibited cytotoxic effects against various human cell lines, including pancreatic, breast, bladder and lung cell lines [110]. Apratoxin A, a cyclic depsipeptide, exerted cytotoxic effects against human HeLa cervical carcinoma cells via cell cycle inhibition [181]. A similar mechanistic effect was reported for the cyclic depsipeptide coibamide A, isolated from *Leptolyngbya* sp. [100], and lyngbyabellin B, isolated from *Lyngbya majuscule* [182]. The linear pentapeptides dolastatin 10 and symplostatin 1 were isolated from *Symploca* spp. and exhibited cytotoxic effects against human lung and breast cancer cell lines, via both Bcl-2 phosphorylation and caspase-3 protein activation [183,184]. In addition, several different types of active peptides have been isolated from *Lyngbya* spp. and *Nostoc* spp., exhibiting anti-proliferative effects via microfilament disruption, secretory pathway inhibition and other intracellular mechanisms [185]. Two novel cyclodepsipeptides, namely, scopularide A and B, isolated from the marine fungus *Scopulariopsis brevicaulis* [186], significantly inhibited the growth of pancreatic and colon cancer cell lines. Sansalvamide A is a structurally unique cyclic depsipeptide isolated from various marine fungi. This compound exhibited cytotoxic activities against different carcinomas, i.e., pancreatic, colon, breast and prostate sarcomas, as well as melanoma, representing a promising anticancer therapeutic lead. The exact mechanism of this depsipeptide is unknown, but a recent study showed an interaction between a heat shock protein (HSP90) and client cancer protein in a mammalian cell line. Sansalvamide A binds to the N-middle domain of HSP90 and allosterically inhibits protein complex formation, which is necessary for promotion of tumor growth [187].

## 7. Anticancer Bioactive Antibiotics Derived from Marine Sources

**Figure 8:**
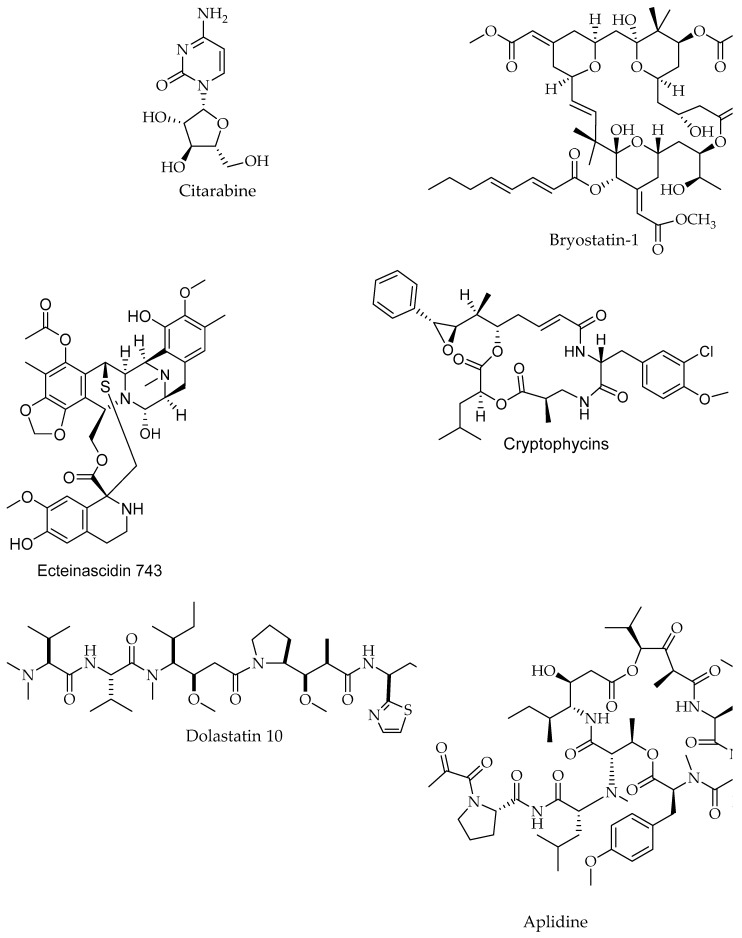

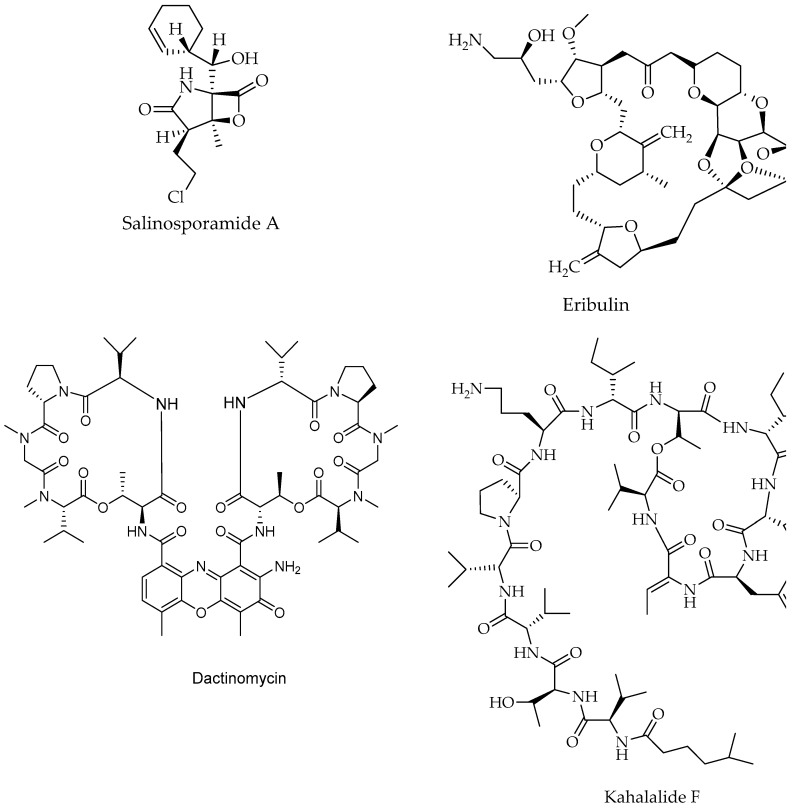
Clinical compounds and drugs from marine organisms used in cancer treatment.

Toxins that originally evolved to kill competing microorganisms can have a variety of physiological effects and could function as novel targets for anticancer drug discovery. In many cases, the targets of these compounds are components of signal transduction cascades that are conserved in many species [213]. Antitumor antibiotics are among the most important cancer chemotherapeutic agents and include members of the anthracycline, actinomycin, and aureolic acid families [19,34,214]. Clinically useful agents from these families (Figure 3, Figure 8; Table 2) include peptolides, dactinomycin, was found to downregulate several glioma metabolic enzymes of glycolysis, glutaminolysis, and lipogenesis, suggesting that targeting multiple tumor metabolic regulators might be a new anti-glioma mechanism of actinomycin D [195]. Anthracyclines are among the most widely used antitumor antibiotics in the clinic and exert antitumor activity mainly by inhibiting topoisomerase II [215,216,217,218,219].

Geldanamycin is a natural fermentation product of the benzoquinone ansamycin and inhibits the heat shock protein HSP90 [220], as well show cytotoxic effect against HeLa cells [221]. Trabectedin and pegylated liposomal doxorubicin have been combined in a randomized phase III study to combat ovarian cancer in vivo and compared to pegylated liposomal doxorubicin alone [222]. The three fused tetrahydroisoquinoline rings contributed to the trabectedin complex mechanism of action. It was claimed to block the substantial DNA and transcription interacting effect as the chemical structure binds to the minor groove of DNA covalently and interact with transcription factors (e.g., SP-1) directly [223]. Another chemotherapeutic agent is bryostatin that modulates the paclitaxel inhibitor of protein kinase C (PKC) [224].

Bryostatin 1, best known anticancer agent isolated from a species of bryozoan *Bugula neritina*, is able to induce ubiquitination and proteasome degradation of Bcl-2 in lymphoblastic leukemia and permits the growth of progenitor cells from bone marrow [225]. Bryostatins are potent activators of protein kinase C (PKC) and regulate the activation, growth, and differentiation of cells [226]. Some of the other suggested mechanism of actions were illustrated and included cell cycle arrest, inhibition of protein synthesis and antiangiogenic activity corresponding to didemnin B and aplidine. Several pathways are proposed for kahalalide F with specific interactions with cell membrane proteins [16]. Aplidine displays promising anti-proliferative activity (currently in phase I–II trials) via characterized delay of neuromuscular toxicity and promising anti-proliferative activity (Figure 8) [227]. Squalamine, neovastat, and LAF389 were investigated for their antiangiogenic activity where squalamine and neovastat are currently tested in phase II and III studies, respectively [16]. Squalamine and LAF389 inhibited sodium hydrogen antiporter sodium-proton exchangers thus targeting the phospholipid bilayer, and with LAF389 entered phase I trial [228]. On the other hand, neovastat stops the binding of VEGF to its receptor [227,229]. Plinabulin (NPI-2358), a potent and selective vascular disrupting agent (VDA) isolated from a marine fungal extract, is presently undergoing phase II clinical trials because of its activity against multi-drug resistant human tumor cell lines [25,101]. Tasidotin, Synthadotin (ILX-651), derived from a marine bacterium in phase II clinical trials, and Soblidotin (TZT 1027), the bacterial peptide of marine origin in phase III clinical trials, are promising anticancer agents [101].

Salinosporamide A, a novel long-lasting proteasome inhibitor isolated from a marine bacterium *Salinispora tropica* [230] in phase I clinical trials, has more efficacy against a wider range of hematologic malignancies and many solid tumor models, and less cytotoxicity to normal cells (Figure 8) [101]. Sorbicillactone-A, is an alkaloid produced by *Penicillium chrysogenum* and associated with marine sponge *Ircinia fasciculate*, showed antileukemic properties [231]. Depsipeptide (NSC 630176), a bicyclic peptide isolated from *Chromobacterium violaceum*, decreased the mRNA expression of the c-MYC oncogene, causing cell-cycle arrest at G0-G1 and acting as an inhibitor of a histone deacetylase [232].

## 8. Chemical Property Space

**Figure 9:**
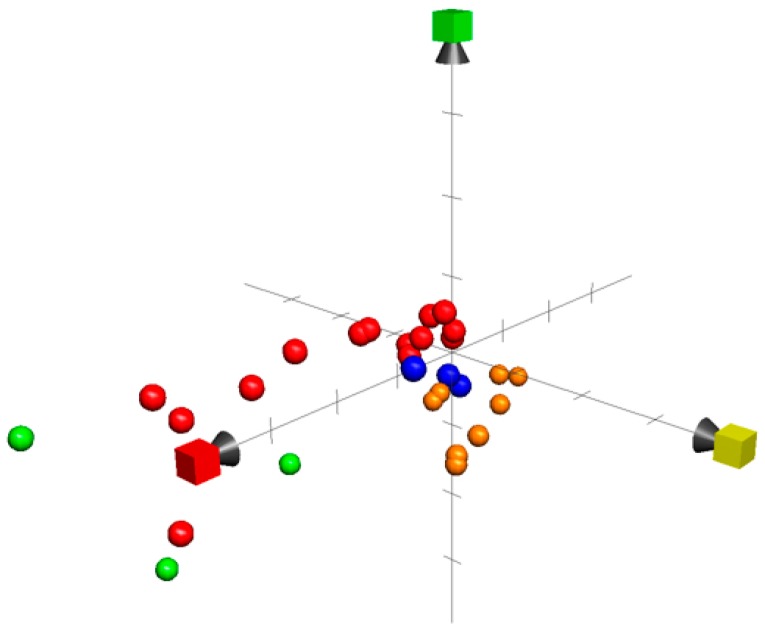
Marine compounds plotted as red, orange, green and blue spheres indicating peptide, polyphenols, polysaccharides and alkaloids respectively. PCs; the red box indicates, PC1, the yellow box indicates, PC2, and the green box indicates, PC3.

The chemical global positioning system of natural products shortly called ChemGPS-NP [233] was used to investigate the chemical property space produced by the marine compounds in this review. The chemical structures were converted to SMILES (simplified molecular-input line-entry system), formats using Chemsketsh [234], and submitted to ChemGPS-NPweb. Eight principle component (PC) values were obtained based on 35 molecular descriptors for each compound. The three major values were plotted on three dimensions graphs as PC1, PC2, and PC3 in order to indicate among others; the size, weight, aromaticity, rigidity, and lipophilicity. Principle component analysis represents the most group of variant properties where every position indicates a compound-specific value in a virtual chemical property space. Figure 9 showed the diversities of studied marine compounds within their respective chemical class as their specific positions did not form a tight cluster in chemical property space. The unique diversity within a single chemical class was noted to be in agreement with Muigg et al 2013 who reported a unique distinction between marine and terrestrial compounds by using chemical property space [235]. For example, marine peptides did spread over and did not form a close cluster forms indicating an interesting diversity within a similar chemical class. The demonstration of marine anticancer compounds as a set of compounds in term of chemical property space can be used to compare different activities using other specific set or databases. Natural marine compounds are no exception from the natural compounds that continue to be a source of unique diversity even by their chemical-property.

## 9. Conclusions

Several marine natural products have been found to exhibit anticancer activity in vitro on a wide range of tumor cell lines, including renal, lung, prostate, bladder, melanoma, osteosarcoma, mammary, and lymphoid cancer-derived cell lines. In addition, most of the reports on the mechanism of action of marine products in inhibiting tumor growth both in vitro and in vivo suggest it is mediated via apoptosis, necrosis, and lysis of the tumor cells. Marine flora, including microalgae, fungi, seaweeds, mangroves, bacteria, cyanobacteria, actinobacteria, and halophytes were found to have anticancer activity in in vitro and in vivo models are extremely important oceanic resources. In this context, reports on the bioactive molecules combating a wide range of tumor cells such as prostate, bladder, renal, lung, mammary, melanoma, bone, and blood cancers, together with the known knowledge of the mechanism of action mediated via necrosis, apoptosis, and tumor cells lysis were discussed herein to illustrate the medicinally potent chemicals associated with the discovery of new anti-cancer drugs. Polyphenols, polysaccharides, alkaloids, peptides, and terpenoids (cembranoids) are some of the potential marine organism metabolites (Table 2), that exhibited an array of antioxidant, antitumor activities, in addition to immunostimulation. The technological innovation and scientific advances provided a baseline for exploring a great scope of the chemically unique, biologically active, and taxonomically diverse marine floras. Eribulin, trabectedin, cytarabine, and brentuximab vedotin, derived from marine resources, are some of the successful examples that predominantly have proven effective in preventing oxidative damage of DNA, induce apoptosis, control carcinogenesis and activate macrophages in pre-clinical and clinical trials. In this review, we summarized the contributions of marine natural products to treat cancer via modulation of cancer-related factors involving oxidative stress, inflammation, and cell survival. We discussed the pharmaceutical prospects and the chemical space properties that provided crucial insights and valuable knowledge on the largely unexplored marine flora-based anticancer leads. Although more detailed investigations are essential to meet the most common challenges of the clinical utility, it is clear that marine products are promising in providing a platform for improving the anti-cancer therapeutic strategies.

## Figures and Tables

**Table 1:** List of compounds isolated from marine sources with potential anticancer effect.

Compound Name/Class	Marine Source	Type of Cancer	Mechanism	References
Apratoxin A/Peptide	*Lyngbya boulloni*, bacteria	Cervical cancer	Cell cycle inhibition IC_50_ = 2.2 nM	[181]
Brugine/Alkaloid	*Bruguiera sexangula*, plant	Sarcoma 180 and Lewis	Not reported	[31]
Fucoidan/Polysaccharides	*Ascophyllum nodosum*, algea	Colon cancer	Inhibit the proliferation of arterial smooth muscle cells at conc. of 80 to 100 µg/mL	[78]
Lyngbyabellin B/p Peptide	*Lyngbya majuscule, bacteria*	Burkitt lymphoma cancer	Inhibit of cell growth IC_50_ = 0.02 µM	[182]
Sansalvamide A/Peptide	Marine fungi	Pancreatic, colon, breast and prostate cancers	Inhibits protein complex formation	[187]
Scutellarein 4′-methylether/Polyphenol	*Osmundea pinnatifida*, algea	Choriocarcinoma cancer	Not reported	[158,188]
Phlorofucofuroecol A/Polyphenol	Brown seaweeds	Cancer	Not reported	[189]
Phloroglucinol/polyphenol	Brown seaweed	Colon cancer	Induce DNA damage, and cell death at 300 µM	[190]
Heparin/Heparan/Ppolysaccharides	*Dictyopteris delicatula, Seaweed*	Colon cancer	Inhibit the proliferation of arterial smooth muscle cells at 80 to 100 µg/mL	[78,191]
Chondroitin-4-sulphate/Polysaccharides	*Cucumaria frondosa*, sea cucumber		Not reported	[31,192]
Chondroitin-6-sulphate/Polysaccharides	*Cucumaria frondosa*, sea cucumber		Not reported	[31,192]

**Table 2:** List of clinical compounds and natural products isolated from marine sources with potential anticancer effect.

Compound Name/Chemical Class	Marine Source	Type of Cancer	Mechanism	Clinical Status/Study Type	References
AE-941/Peptide	Shark cartilage	Renal, lung cancer	Inhibition of gelatinolytic and elastinolytic activities of MMP-2, MMP-9, and MMP-12. The MMP’s are often over expressed in tumors and play an important role in the degradation of the (extracellular matrix allowing tumor growth and invasion (metastasis)	Drug, phase 3, Investigationa, Interventional	[44,193,194]
Actinomycin/Peptide	*Streptomyces parvullus*, *Streptomyces* sp. ZZ338 Actinomyces	Childhood cancer, Wilms tumor	Inhibition of RNA polymerase	Drug, phase 4, Interventional	[195,196,197]
Aplidine (Plitidepsin, Dehydrodide-mnin B)/Peptide	*Aplidium albicans*, Tunicate, Ascidiacea	Pancreatic, stomach, bladder, and prostate cancers	Activation of protein kinase C	Drug Investigational	[198,199]
		Leukemia Non Hodgkin Lymphoma	Induce the apoptotic cascade	Drug phase 2, Interventional	[44,198,200]
Bryostatin-1/Polyketide	*Bugula neritina*, Bryozoa	Metastatic solid tumors	Inhibition of growth and alteration of differentiation	Drug phase 1, 2 Interventional	[44,198,201]
Citarabine/Alkaloid	Sponge	Leukemia (acute non-lymphoblastic)	Inhibition of DNA synthesis	Drug Approved, Investigational	[198,202]
Cryptophycins/Peptide	*Nostoc* sp., Macroalgae & *Dysidea arenaria*, Sponge	Not reported	Tubulin (inhibition of polymerization of microtuble)	Phase 1	[44]
Dolastatin 10/Peptide	*Dolabella auricularia*, Mollusc	Pancreatic cancer	Inhibition of microtubules and pro-apoptotic effects	Drug phase 2, Interventional	[44,198,203]
ET-743 (Trabectedin, Ecteinascidin)/Alkaloid	*Carribean tunicate Ecteinascidia turbinate* Tunicate, Ascidiacea	Sarcomas and ovarian cancer	Binding to the minor groove of DNA interfering with cell division and genetic transcription processes and DNA repair machinery	Drug Approved, Investigational	[44,198,204]
		Breast cancer	Alkylation ofguanine residues in the DNA minor groove	Drug phase 2, Interventional	[198,205]
Eribulin (E7389)/Polyketide	*Lissodendoryx* sp. *Halichondria okadai*., Sponge	Breast cancer	Activation of cellular apoptosis under anchorage-independent and -dependent cell culture conditions	Phase 1,2, Investigationa, Interventional	[44,206,207]
		Advanced solid tumors, breast	Inhibition of growth phase of microtubules without affecting the shortening phase and sequesters tubulin into nonproductive aggregates	Drug, Approved, phase 2, Investigationa, Interventional	[208,209]
Kahalalide F/Peptide	*Elysia rufescens*, Mollusc/*Bryopsis* sp., Macroalgae	Prostate cancer	Induction of changes in lysosomal membrane	Phase 2	[44]
PM02734/Peptide	*Elysia rufescens*, Mollusk	Breast, colon, pancreas, lung and prostate	Antiproliferative	Drug, phase 1, Investigation, Interventional	[210,211]
Salinosporamide A (Marizomib^®^) (NPI-0052)/Polyketide	*Salinospora tropica*, actinomyces		Prevention of proteins breakdown involved in signal transduction, which blocks the cancer cells growth and survival	Drug phase 1, Interventional	[17,44,212]
